# Tertiary cytoreduction for recurrence of ovarian CANCER patients after intraperitoneal chemotherapy

**DOI:** 10.3389/fmed.2025.1691155

**Published:** 2025-11-28

**Authors:** Giusi Santangelo, Enrico Ciminello, Tullio Golia d’Augè, Violante Di Donato, Federica Tomao, Innocenza Palaia, Margherita Fischetti, Giorgio Bogani, Ludovico Muzii, Pierluigi Benedetti Panici, Giorgia Perniola

**Affiliations:** 1 Department of Gynecological, Italy – Policlinico Umberto I, Viale Regina Elena, Obstetrical and Urological Sciences, “Sapienza” University of Rome, Rome, Italy; 2 Italian National Institute of Health, Rome, Italy; 3 Gynecologic Oncology Unit, Fondazione IRCCS Istituto Nazionale dei Tumori di Milano, Milan, Italy

**Keywords:** recurrent ovarian cancer, intraperitoneal chemotherapy, intravenous chemotherapy, tertiary cytoreduction, adhesion score

## Abstract

**Introduction:**

This study evaluated the feasibility, complications, and outcomes of a tertiary cytoreduction after second-line treatment with IP (intraperitoneal) versus IV (intravenous) chemotherapy in recurrent ovarian cancer (ROC) patients.

**Methods:**

We retrospectively collected data of patients treated with an optimal tertiary cytoreduction. At the second relapse, the patients underwent optimal secondary surgery followed by IP chemotherapy (case group) or by IV chemotherapy (control group). Differences in treatment-related morbidity rate, pattern of recurrence, and oncologic outcomes were evaluated by Mann–Whitney and Chi-Squared. Kaplan–Meier and frailty model for recurrent events were used to assess statistical significance in differences of disease-free survival.

**Results:**

Charts of 60 patients with a second ROC were identified. The patients with extensive peritoneal carcinomatosis or an inaccessible abdominal cavity were excluded. Twenty-nine patients (48.3%) who underwent optimal tertiary cytoreduction were included: 16 and 13 patients were from the IP and IV groups, respectively. At the second relapse, 56.2% of patients in the IP group and 61.5% in the IV group presented oligometastatic disease, respectively. The adhesions were significantly more represented in the IP group than the IV group (*p* = 0.01). Days to the first flatus were significantly different in the two groups (4.2 in IP group and 2.5 days in IV group, *p* < 0.01).

**Conclusion:**

The present study showed that IP chemotherapy does not represent an obstacle to surgery in ROC patients. The surgery after IP is feasible. No significant differences in terms of complications and outcomes were observed in the two groups.

## 1 Introduction

Epithelial ovarian cancer (OC) is the deadliest of gynecologic malignancies, with an estimated 20,890 new cases diagnosed during 2025 in the world, and 12,730 deaths (1).

Surgery with complete macroscopic resection and platinum-based chemotherapy represent the cornerstones of the treatment in the primary setting of ovarian cancer (2–4). This approach is also strongly considered for patients with recurrent disease. DESKTOP III (5), SOC 1 (6), and GOG 0213 (7) trials showed the importance of secondary cytoreduction as a treatment option for well-selected patients, achieving a longer overall survival than chemotherapy alone (8–10). Likewise, tertiary cytoreductive surgery data showed a survival benefit in a highly selected group of patients in whom a complete gross resection can be achieved (11–13). Manning-Geist et al., in a large study of 114 women at tertiary cytoreduction showed that the complete gross resection, the treatment-free interval, and a platinum sensitivity were all significantly associated with disease-specific survival (DSS) and maintained significance on the multivariate analysis (HR 3.71, 95% CI: 1.59–8.70; HR 0.49, 95% CI: 0.28–0.85; and HR 2.94, 95% CI: 1.22–7.07, respectively) (14). The adjuvant treatment of ROC mainly comprises various combinations of systemic chemotherapy with or without targeted agents. In the past 20 years, intra-peritoneal chemotherapy was introduced in clinical practice. This route has revealed a survival benefit in the primary treatment setting (15–18). Following these good results, some authors experimented the IP chemotherapy as an adjuvant treatment in recurrent ovarian cancer (19).

Since the mid-1990s, several trials have demonstrated superior survival when chemotherapy is delivered intraperitoneally (IP) (11, 14, 15, 19). A phase III randomized controlled trial (RCT)—GOG 172—comparing IV and IP chemotherapy confirmed significantly longer progression-free survival (PFS) and overall survival (OS) with the IP regimen (16). The median PFS for IV and IP chemotherapy was 18.3 and 23.8 months, respectively, and the median OS was 49.7 and 65.6 months, respectively.

Markman et al. conducted a meta-analysis of seven GOG phase II studies about second-line IP chemotherapy to evaluate survival, achieving a median OS of 2.4 years and a DFI of 1.2 years (20).

Several factors have been identified as potential barriers to the integration of IP/IV chemotherapy into practice (21, 22), including treatment-related toxicities (17), the absence of a standard regimen, and catheter-related complications (22). Since the early 2000s, Benedetti Panici et al. proposed the IP-chemotherapy infusion by ultrasound-guided direct puncture to reduce the risk of catheter-related complications and decrease IP-chemotherapy discontinuation (23).

We aim to assess if IP chemotherapy could represent an obstacle to recurrence surgery for the occurrence of multiple adhesions, fibrosis, collections of fluid, and weakened tissues. This study evaluated the feasibility, complications, and outcomes of the tertiary cytoreduction after IP (intraperitoneal) chemotherapy and evaluated the type of recurrence (single, multiple, carcinomatosis, and site of localization) compared with a group of ROC patients previously treated only with IV (intravenous) agents.

## 2 Materials and methods

### 2.1 Patient population

This is a retrospective, single-institution study collecting data of women who underwent tertiary cytoreduction after IP chemotherapy or IV chemotherapy as adjuvant treatment for primary recurrence, between May 01, 2008, and July 30, 2020, at the Department of Maternal Child and Urological Sciences, of Policlinic Umberto I, “Sapienza” University of Rome. Written informed consent was obtained from all patients. All data were retrospectively evaluated from a prospectively collected clinical database. The patient’s characteristics were evaluated for age, tumor histology, tumor grade, stage at diagnosis, and performance status (PS).

Eligibility criteria were:

1. histologically or cytologically confirmed recurrent epithelial ovarian cancer;
2. Performance Status between 0 and 1 according to Eastern Cooperative Oncology Group (ECOG); (3) Optimal primary and secondary cytoreductive surgery (RT = 0) followed by adjuvant platinum-based chemotherapy;
3. Disease-free survival from the end of the previous line of chemotherapy >6 months;
4. Evidence of intra-abdominal disease at radiologic evaluation by computed tomography (CT scan) or at positron emission tomography/computed tomography (PET-CT scan).

Eligible patients required appropriate hematologic, renal, and hepatic functions.

Exclusion criteria were represented by previous abdominal radiotherapy.

In order to verify the clinical and survival implications of IP chemotherapy, a historical control group was identified between May 01, 2008, and July 30, 2020. The control group consisted of 50 consecutively selected patients with platinum-sensitive recurrent ovarian cancer (ROC), retrospectively identified and treated with intravenous (IV) chemotherapy at our institution. Data were retrieved from the patients’ charts. Patients were assigned to ensure homogeneous baseline characteristics between groups and minimize confounding factors. The same team treated both target (IP) and control (IV) groups.

At initial diagnosis, all patients underwent optimal cytoreductive surgery, achieving no residual disease (RT = 0), followed by platinum-based chemotherapy. At the time of first recurrence, all patients again received optimal secondary cytoreductive surgery (RT = 0), followed by intravenous (IV) or intraperitoneal (IP) platinum-based chemotherapy. The choice of second line medical treatment based on patient’s physical characteristics and chemotherapy tolerance.

Collected surgical data included:

- type of surgery (diagnostic laparoscopy, explorative laparotomy, operative laparotomy),
- type of recurrence (carcinomatosis, single, multiple),
- sites of recurrence,
- residual tumor (RT) after surgery
- adhesions score.

Adhesions score indicated 0 for no adhesions, 1 for filmy adhesions, blunt dissection, 2 for strong adhesions, sharp dissection, 3 for very strong vascularized adhesions, sharp dissection, damage hardly preventable and 4 for inaccessible peritoneum (24).

Sample size was determined as being at least equal to the minimal one ensuring an 80% power and 95% statistical significance in detecting a 12 months (1 year) difference in time to relapse after IP/IV treatment with standard deviation in target outcome assumed to be equal to 10 (*n* = 11 per arm).

### 2.2 Peri-operative data

Data of interest included median operative time (minutes), intra- and post-operative complications, pre-and post-operative hemoglobin (Hb) levels, bowel first flatus, length of hospital stay (days from surgery to discharge), admission to intensive care units (days), number of surgical procedure and type of surgery (splenectomy, lymphadenectomy, resection of diaphragm metastasis and bowel resection).

### 2.3 Outcome measures

Our coprimary outcome measures were difference in patients previously treated with IP vs. IV in terms of surgical features type of recurrence, sites of recurrence, residual tumor (RT) after surgery, adhesions score and outcomes (PFS and OS).

- DFI1 was identified as the time from the end of first ovarian cancer treatment (diagnosis/first line of chemotherapy) to first ovarian cancer relapse.
- DFI2 was identified as the time from the end of treatment for first relapse to second relapse (DFI2).

Progression Free Survival (PFS) was calculated from the end of treatment for first relapse (IP or IV) to second relapse.

Overall Survival (OS) was calculated from the end of treatment for first relapse (IP or IV) to death of patients based on treatment groups.

The median follow up duration was of 79 months.

### 2.4 Statistical analyses

Descriptive data were reported in terms of mean (standard deviation) and counts (percentage) for continuous and categorical variables, respectively. The statistical significance of the differences between the two groups was investigated via Mann–Whitney test for unpaired samples for continuous variables and by χ2 test for categorical ones. Crude survival rates with 95% Confidence Intervals (CI_95%_) were estimated by Kaplan–Meier with secondary cytoreduction and death as target events; the statistical significance in survival differences between the two groups was explored by log-rank test. Frailty model for failure time in the presence of recurrent events (25) was used to evaluate the impact of the treatment on disease-free and overall survival, measured by hazard ratio, both unadjusted and adjusted for baseline characteristics. The threshold of statistical significance is fixed equal to 0.05 for all *p*-values. Statistical analyses were performed by using the software R version 4.2.3 (2023-03-15 ucrt)—“Shortstop Beagle.”

## 3 Results

Data from 29 patients were collected, 13 treated with IV chemotherapy and 16 with IP chemotherapy at first recurrence. The baseline characteristics at the time of primary diagnosis of ovarian cancer are shown in Table 1.

**Table 1:** Baseline characteristics.

	IV *n* = 13	IP *n* = 16	*p*-value
Age	55.9 (12.9)	51.9 (12.6)	0.32
Laparoscopy	5 (38.5%)	5 (31.2%)	0.99
NACT	5 (38.5%)	3 (18.8%)	0.45
Histotype			
Endometroid	1 (7.7%)	1 (6.2%)	0.65
Mucinous	0 (0%)	1 (6.2%)	
Serous	12 (92.3%)	14 (87.5%)	
Grade			
Grade 2	2 (15.4%)	2 (12.5%)	1
Grade 3	11 (84.6%)	14 (87.5%)	
Stage			
FIGO stage IIB	2 (15.4%)	1 (6.2%)	0.52
FIGO stage IIIB	3 (23.1%)	3 (18.8%)	
FIGO stage IIIC	8 (61.5%)	10 (62.5%)	
FIGO stage IVB	0 (0%)	2 (12.5%)	

NACT, neoadjuvant chemotherapy.

Mean age was 55.9 years in the IV group and 51.9 years in the IP group. Papillary serous adenocarcinoma was the most common histologic finding (92.3% in IV group and 87.5% in IP group), and the primary tumors were more often grade 3 (84.6% in IV group and 87.5% in IP group). Most of the patients presented at diagnosis an advanced stage (stage IIIB-IV): 84.6% of IV group and 93.8% of IP group.

Table 2 shows the characteristics at first relapse in terms of localization and number of metastases. The complication rate and the number of procedures performed during secondary cytoreductive surgery were also reported. No statistically significant difference between the two groups’ baseline features was detected (Table 2).

**Table 2:** First relapse information DFI 1: disease free interval (time to first surgery to first relapse).

	IV (mean +SD)	IP (mean +SD)	*p*-value
DFI 1 (months)	21 (14.7)	23.4 (23.1)	0.08
Localization relapse			
Upper abdomen	7 (53.8%)	9 (56.2%)	0.14
Lymph node	1 (7.7%)	5 (31.2%)	
Pelvic	5 (38.5%)	2 (12.5%)	
N. of lesion			
Single lesion	11 (84.6%)	10 (62.5%)	0.36
Multiple lesion	2 (15.4%)	6 (37.5%)	
N. procedure men	2 (2.2)	1.4 (1)	0.67
30 days complications	1 (7.7%)	1 (6.2%)	1

The IV group patients had on average 21 (SD = 14.7) months of disease-free time (DFI1), while patients in the IP group after primary surgery were disease free for 23.4 (SD = 23.1) months on average (*p* = 0.08).

The mean disease-free interval (DFI2) between secondary cytoreduction (for first recurrence) and the onset of a second relapse was 11.8 months (SD = 5.5) in patients treated with intravenous chemotherapy and 15.1 months (SD = 15.2) in those treated with intraperitoneal chemotherapy (*p* = 0.96). No statistically significant difference was observed between the two groups receiving second-line IV or IP chemotherapy.

At the tertiary cytoreduction, a higher number of adhesions were observed in IP patients (93.8%) than IV patients (46.2%) (*p* < 0.05).

At the tertiary cytoreduction, the canalization days were statistically different in the two groups (2.5 days in IV group and 3.7 days in IP group, *p* < 0.01) (Tables 3, 4).

**Table 3:** Second relapse information.

	IV *n* = 13	IP *n* = 16	*p*-value
DFI 2	11.8 (5.5)	15.1 (15.2)	0.96
Local/pelvic	9 (69.2%)	10 (62.5%)	1
Lymph node	4 (30.8%)	6 (37.5%)	
Single lesion	8 (61.5%)	9 (56.2%)	1
Multiple lesion	5 (38.5%)	7 (43.8%)	
Adhesion	6 (46.2%)	15 (93.8%)	**0.01**
Adhesion score	1.5 (1.8)	1.9 (1)	0.33
N. procedure	2.2 (1)	1.7 (0.7)	0.72
30 days complications	3 (23.1%)	1 (6.2%)	0.44

DFI 2: disease free interval 2(time to second surgery to second relapse).

**Table 4:** Tertiary cytoreduction information.

	IV *n* = 13	IP *n* = 16	*p*-value
Hb post-operative	11.4 (1.1)	11.1 (1)	0.36
Surgery time (minutes)	221.5 (111.3)	178.1 (69)	0.3
Intensive Unit (days)	3 (23.1)	0 (0)	0.16
Canalization (days)	2.5 (0.8)	4.2 (1.4)	**<0.01**
Hospital stay (days)	10 (4.3)	7.9 (5.2)	0.1

Hb, Hemoglobin.

**Figure 1:**
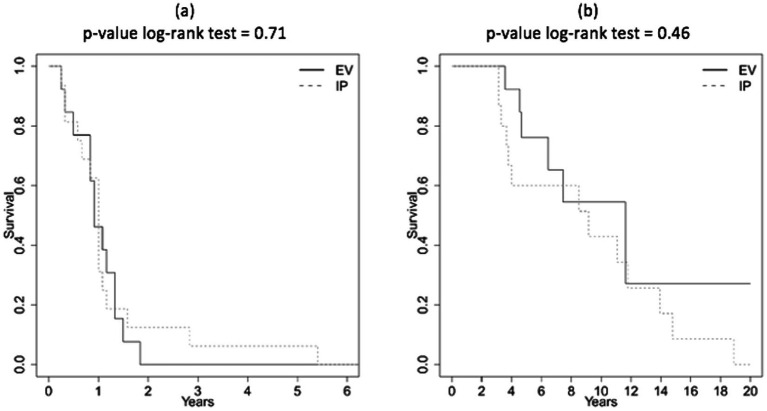
**(a)** Form treatment to second relapse (PFS). **(b)** Form treatment to death (OS).

The estimated crude survival curves from IP/IV treatment for primary recurrence and death as the target event are shown in Figure 1. The crude survival at 6 months for PFS is equal to 81.2% (CI_95%_: 64.2–100%) for IP group and to 76.9% (CI_95%_: 57.1–100%) for IV group, while the crude survival for PFS at 12 months is equal to 31.2% (CI_95%_: 15.1–64.6%) for IP group and to 46.1% (CI_95%_: 25.7–83%) for IV group. The crude survival at 5 years for OS is equal to 60% (CI_95%_: 39.7–90.7%) for IP group and to 76.2% (CI_95%_: 55.8–100%) for IV group, while the crude survival at 10 years is equal to 42.9% (CI_95%_: 22.9–80%) for IP group and to 54.4% (CI_95%_: 31–95.4%) for IV group. The difference in crude survival between IP and IV was not significant according to the log-rank test for both PFS and OS.

Table 5 reports the impact of treating patients by IP (IV treatment as baseline) after secondary cytoreduction on disease-free and overall survival time for recurrent events when secondary relapse were taken into account. Both models, unadjusted and adjusted for baseline characteristics, did not show statistically significant differences in outcomes depending on the choice of treatment (26).

**Table 5:** Estimated impact of IP on disease and overall survival.

	HR	Log-HR	Standard error	*p*-value
Unadjusted model				
Disease free survival	1.02	0.02	0.2	0.32
Overall survival	1.5	0.4	0.4	0.34
Adjusted model for primary characteristics				
Disease free survival	1.1	0.1	0.3	0.84
Overall survival	1.3	0.2	0.4	0.51

## 4 Discussion

Our results demonstrated a significant difference in terms of adhesion score and canalization days between the two groups.

Instead, there wasn’t difference between the two groups for other end points in terms of complications, pattern of recurrence and oncological outcomes (OS and PFS).

In the literature, there aren’t data on the feasibility and complication rate of a tertiary cytoreduction in a cohort of patients who underwent IP chemotherapy at the first relapse of ovarian cancer.

Our study showed that IP chemotherapy does not represent an obstacle to the tertiary cytoreduction, the procedure is feasible and no significant differences in terms of complications and outcomes were observed between the IP group and the IV group. At the time of second recurrence following targeted treatment (IV or IP chemotherapy), no significant differences were observed between the two groups in terms of DFI or recurrence site (pelvic or lymph node).

In the upper abdomen in both groups, we saw a homogeneous distribution of disease, lymph nodal metastasis was more frequent in patients treated with intraperitoneal chemotherapy, on the contrary the number of pelvic relapses was greater in the group of patients treated with intravenous chemotherapy.

Patients treated with intravenous chemotherapy presented more oligometastatic disease than patients treated with IP.

The number of lesions at second recurrence did not reach a statistically significant difference.

Maybe the statistical difference was not reached in terms of the number of lesions and the localization of relapse for the low number of patients enrolled in the study.

Even at the second recurrence, the rate of adhesions was statistically significant in patients treated with IP (*p* = 0.01) and the days of canalization were lower in patients treated with IV (*p* < 0.01), probably due to the lower number of adhesions.

Our study provides real-world data that analyzes the feasibility and the rate of surgery complications after intraperitoneal administration of chemotherapy.

In previous studies, the IP chemotherapy administration showed an improvement in OS (HR 0.79, 95% CI 0.67–0.92) and PFS (HR 0.88, 95% CI 0.80–0.98) over IV-CHT in advanced OC management (15). Nevertheless, survival data from OV21/PETROC and GOG 252 studies are still immature, and therefore, the results should be considered with caution; however, these papers showed that the IP carboplatin-based regimen was well tolerated without reduction in QoL or increase in toxicity compared with IV administration alone (25, 27, 28).

Overall, accumulated data over the years from Phase I and II clinical trials (29, 30), and Phase III randomized studies (30) and a Cochrane review (31) that included 2026 women have demonstrated. significantly longer PFS and OS with IP chemotherapy, and many smaller studies of real-world. single institution reports (such as this study) have demonstrated that IP cisplatin plus paclitaxel-based. chemotherapy in the first-line treatment of ovarian cancer patients with small-volume residual disease is associated with significantly better long-term outcomes. The plausible scientific rationale for this degree of benefit may be that OC remains largely confined to the peritoneal cavity, providing the opportunity to increase the concentration of agents that are cleared slowly from the peritoneal cavity and rapidly from the systemic circulation. For cisplatin, the IP route delivers 10- to 20-fold greater exposure over that achievable with the IV route. Paclitaxel, a large, water-insoluble molecule, has more than an 800-fold increase in the peritoneal cavity compared with the plasma drug level as measured by the area under the concentration– time curve.

Despite the accumulated evidence demonstrating a survival benefit with IP in women with advanced ovarian cancer, and cost-effectiveness studies demonstrating considerable value if it is adopted widely.

Furthermore, preclinical studies suggested that cisplatin is capable of penetrating small-volume tumors (1–3 mm) (15, 32–37).

Interest in chemotherapy administered intraperitoneally as a strategy for reducing the risk of disease recurrence and prolonging survival emerged approximately 30 years ago. Several randomized phase III studies showed an improvement in survival for the combination of delivery of chemotherapy intraperitoneally and intravenously over chemotherapy administered intravenously alone for select patients following primary cytoreductive surgery (38–42).

GOG 172, a randomized phase III study of cisplatin administered intraperitoneally combined with both delivery of paclitaxel intraperitoneally and intravenously, published in 2006, demonstrated a 16-month improvement in median OS over intravenous administration of the same drugs alone (16). This prompted the National Cancer Institute (NCI) to issue a rare clinical announcement regarding the clinical utility of cisplatin-based chemotherapy administered intraperitoneally in the treatment of patients with small volume (<1 cm), advanced-stage (stage III) epithelial ovarian cancer following an attempt at maximal cytoreductive surgery. On average, intraperitoneal/intravenous chemotherapy was associated with a 21.6% decrease in risk of death [hazard ratio (HR) 0.78; 95% CI, 0.69–0.89] (17).

An update published in 2015 with a median follow-up of 10.7 years showed that women who underwent intraperitoneal/intravenous chemotherapy in GOG 172 continued to derive benefit with a median survival of 61.8 months (95% CI, 55.5–69.5 months) compared with 51.4 months (95% CI, 46–58.2 months) for chemotherapy administered intravenously alone (36). The recent Cochrane Review, restricted to newly diagnosed patients receiving treatment after primary cytoreductive surgery, accepted data from eight randomized studies on 2,026 women and concluded that women experienced increased survival if they received intraperitoneal/intravenous chemotherapy (HR 0.81; 95% CI, 0.70–0.9) and that intraperitoneal/ intravenous chemotherapy also prolonged the disease-free interval (five studies, 1,311 women; HR 0.78; 95% CI, 0.7–0.86) (15) thus potentially affecting quality of life going forward.

Despite many years of research, it appears that we have collectively failed to describe the key biologic targets of IP therapy in terms of direct tumor cytotoxicity, alterations in the peritoneal stromal microenvironment (such as a reduction in angiogenesis or growth factors), or enhancement of the host immune response. Each of these pathways is a potential mechanism of clinical benefit for IP cytotoxic chemotherapy (30).

This study offers valuable real-world evidence on the impact of IP chemotherapy on clinical, surgical, and outcome data. The consistent application of inclusion criteria and centralized data collection lends strength to the reliability of findings.

However, this study has several limitations inherent to its retrospective, single-institution design. First, the relatively small sample size (*n* = 29) limits the statistical power to detect differences between groups. This may explain why differences in PFS and OS did not always reach statistical significance.

Second, treatment allocation was non-randomized and was influenced by the treating gynecologic oncologist’s preference, which introduces selection bias. Although baseline characteristics were balanced, unmeasured confounders such as physician experience, patient preferences, or subtle surgical factors may have influenced outcomes.

Third, the choice of chemotherapy regimen evolved over time, with earlier patients more likely to receive IP chemotherapy and later patients receiving dose-dense IV regimens. This temporal bias may be associated with changes in supportive care, surgical techniques, or institutional practices that could confound survival outcomes.

Overall, intraperitoneal chemotherapy represents an important tool to optimize local control, reduce recurrence-related morbidity, and personalize treatment in women with recurrent ovarian cancer, contributing to improved clinical outcomes and quality of life.

Although the study demonstrates the feasibility of surgery after IP chemotherapy in real-world settings, its generalizability may be limited, as institutional resources, surgical expertise, and patient counseling infrastructure may not be universally available or evenly distributed.

So, the future prospectives will see to personalized therapy with delivery technologies that increase efficacy and reduce toxicity, and more patient-friendly delivery.

## 5 Conclusion

In conclusion, this study on a small patient population treated at a single institution provides strong evidence that IP chemotherapy does not represent an obstacle to subsequent surgical treatment and is not associated with worse overall survival (OS) and PFS outcomes in women with ovarian cancer. Eligible patients may benefit from being offered this option, along with counseling and support.

The sample size is a limit of our study and other study should be made in multicentric, prospective, randomized way to could reach a consistent scientific evidence.

Now more than ever, we have novel combinations to personalize upfront treatments for advanced ovarian cancer. In addition to IP therapy, we also need to focus on targeted therapy, bio- markers, survivorship, and the sequencing of therapy (43, 44).

IP chemotherapy represents a valid chance for patients with peritoneal carcinomatosis and refractory ascites, especially in patients in whom paracentesis was performed for improved breath. Nowadays, with the coming of target therapy, maybe IP has become the last chance, but it is a valid chance not to be forgotten.

## Data Availability

The original contributions presented in the study are included in the article/supplementary material, further inquiries can be directed to the corresponding author.
